# Comparative genomics of planktonic *Flavobacteriaceae* from the Gulf of Maine using metagenomic data

**DOI:** 10.1186/2049-2618-2-34

**Published:** 2014-09-05

**Authors:** Benjamin J Tully, Rohan Sachdeva, Karla B Heidelberg, John F Heidelberg

**Affiliations:** 1 Biological Science, Marine & Environmental Biology, Dornsife College of Letters, Arts and Sciences, University of Southern California, 3616 Trousdale Parkway, Los Angeles CA 90089, USA

**Keywords:** Metagenomics, Microbial ecology, Comparative genomics

## Abstract

**Background:**

The Gulf of Maine is an important biological province of the Northwest Atlantic with high productivity year round. From an environmental Sanger-based metagenome, sampled in summer and winter, we were able to assemble and explore the partial environmental genomes of uncultured members of the class *Flavobacteria.* Each of the environmental genomes represents organisms that compose less than 1% of the total microbial metagenome.

**Results:**

Four partial environmental genomes were assembled with varying degrees of estimated completeness (37%–84% complete) and were analyzed from a perspective of gathering information regarding niche partitioning between co-occurring organisms. Comparative genomics revealed potentially important niche partitioning genomic variations, including iron transporters and genes associated with cell attachment and polymer degradation. Analysis of large syntenic regions helped reveal potentially ecologically relevant variations for *Flavobacteriaceae* in the Gulf of Maine, such as arginine biosynthesis, and identify a putative genomic island incorporating novel exogenous genes from the environment.

**Conclusions:**

Biogeographic analysis revealed flavobacteria species with distinct abundance patterns suggesting the presence of local blooms relative to the other species, as well as seasonally selected organisms. The analysis of genomic content for the Gulf of Maine *Flavobacteria* supports the hypothesis of a particle-associated lifestyle and specifically highlights a number of putative coding sequences that may play a role in the remineralization of particulate organic matter. And lastly, analysis of the underlying sequences for each assembled genome revealed seasonal and nonseasonal variants of specific genes implicating a dynamic interaction between individuals within the species.

## Background

The *Flavobacteriaceae* have been indicated as major constituents of microbial communities attached to detritus and planktonic organisms [1,2]. Through this interaction, they are presumed to be important players in the “microbial loop”, a trophic model by which dissolved organic matter is consumed by microorganisms that are in turn consumed by other organisms, breaking down large organic molecules (e.g., proteins, chitin, and other polysaccharides) and making them accessible to other microbes [3]. The microbial loop in the surface ocean is estimated to degrade and recycle about 50% of surface primary production [4]. Within the microbial loop, individual species typically play specialized roles in polymeric degradation; frequently, they have specialization in expressed enzymes that target only specific compounds and are limited in the range of compounds [5]. Therefore, a change in the substrates within an environment influences microbial composition [6]. Particle-associated microorganisms thrive in limited, ephemeral niches attached to marine particles, with the plankton possibly acting as a source from which individuals are drawn to compose future assemblages [7].

Metagenomics has previously been used to reconstruct microbial genomic potential and to predict microbial structure from diverse environments [8-12]. Many metagenomic studies involved the reconstruction of single organisms (or species-level populations), linking metabolic functions with phylogeny [8,13,14]. Expanding our understanding of the genomic content of species from the environment is crucial in determining the putative roles these organisms play within in the ecosystem. However, extrapolating 16S rRNA gene sequence similarity to putative functional roles is confounded due to large genomic variations in between related organisms, with as much as 60% dissimilarity in gene content [15,16]. These variations in genomic content play a key role in determining the ecological niche that a given species can occupy and are likely the underpinning of microbial speciation events [17]. To date, a limited number of environmental datasets [18-20] have been used to assess *in situ* population-level variations of microorganisms, combining the analysis comparative genomics of related species that occupy the same spatial and temporal environments and selection pressures acting on individual genes.

The Gulf of Maine (GoMA) is an oceanic province of high biological significance, with a high degree of primary productivity and historically important commercial fisheries [21] (supplemental figures can be found in Additional file 1: Figure S1). From an environmental metagenome from the GoMA, we were able to reconstruct four partial environmental genomes (phylogenetic bins) and explore the functional capacity of Flavobacteria present in the microbial community. As with other genomes from metagenomic source material, these partial genomes represent a composite of all the organisms with similar genotypes within the community [22]. We are able to highlight genomic variations that potentially could allow for each individual *Flavobacteria* to exploit specific microhabitats within the environment resulting in non-overlapping niches. Competitive exclusion is alleviated at the species level or subspecies level when genomic diversification results in decreased competition for limited resources. One possible mechanism by which organisms can alleviate competitive exclusion at the species level or subspecies level is through diversification of genomic content [5]. These sorts of comparative genomic techniques have been predominantly performed on cultured isolates [23,24], and while such analyses have been performed on environmental microbial metagenomic datasets, this comparative genomic analysis is still underrepresented in metagenomic studies compared to studies analyzing total gene content.

## Results and discussion

### Sequencing, community structure, assembly, and binning assessment

Over 2.8 million Sanger sequences from samples collected in January (winter) and August (summer) of 2006 were analyzed (Additional file 1: Figure S1, [14], supplemental tables can be found in Additional file 2: Table S1). In total, 2,238 16S rRNA gene fragments of at least 250 bp in length were identified, of which 2,213 could be classified using the RDP classifier (see supplemental materials and methods in Additional file 3). The *Bacteroidetes* represented the third largest group in both summer and winter (15.0% and 15.6% sequences, respectively), following the Alpha- and Gammaproteobacteria. From within the *Bacteroidetes*, 75 sequences were identified as members of the class *Flavobacteria* (3.4%). When viewed at phyla and class level, most microbial groups showed similar abundances in both summer and winter (Additional file 1: Figure S2). However, this relationship was not maintained as the taxonomic resolution increased. Many of the sub-phyla and sub-class populations showed some seasonal variation (see below).

**Figure 1:**
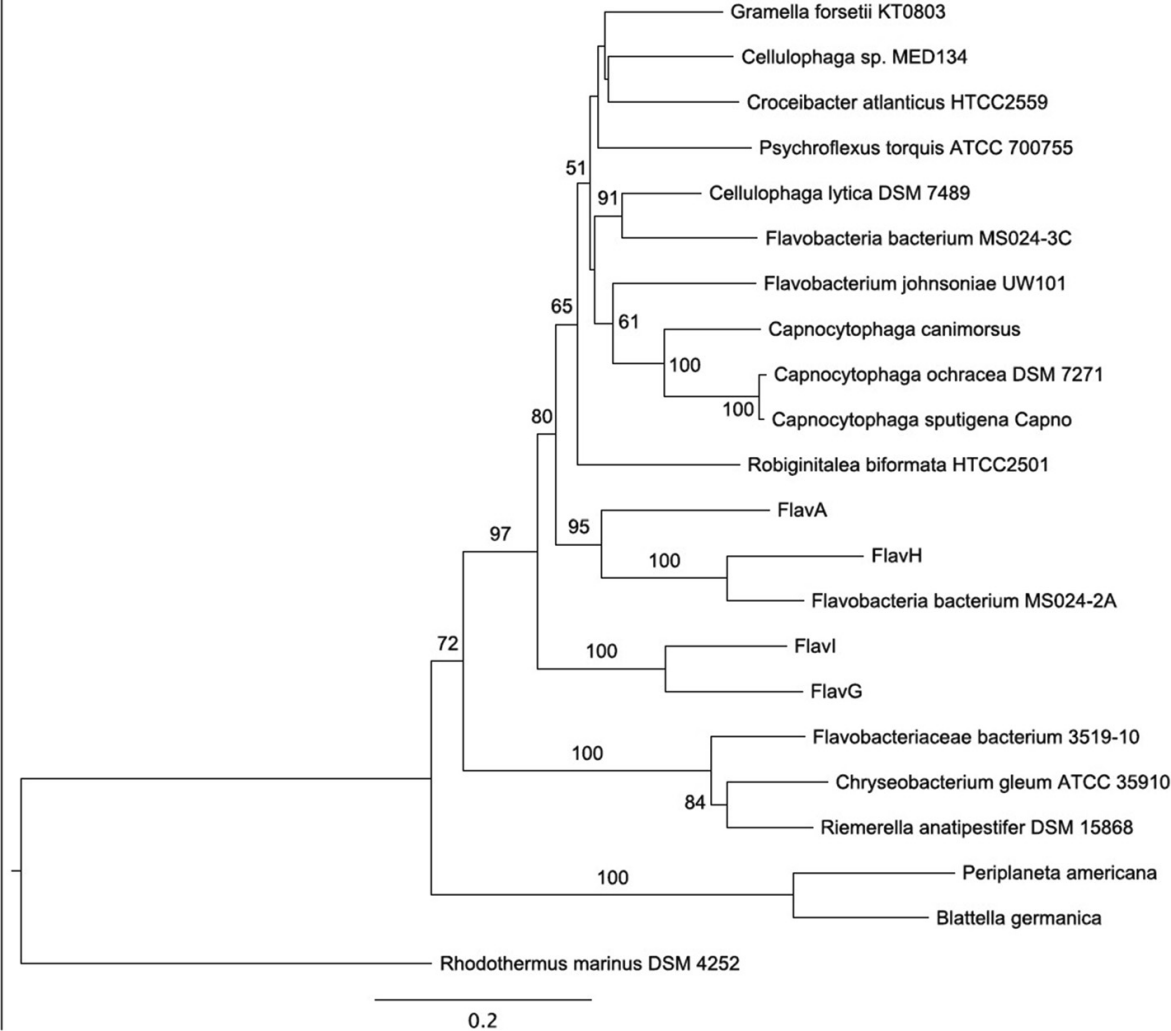
**Maximum likelihood tree generated using PHYML (bootstrap 1,000) of DNA primase (DnaG) identified in the four phylogenetic bins and other members of the class *Flavobacteriaceae *(489 amino acids).** Only bootstrap values greater than 50% are shown.

In brief, the binning and assessment of the putative flavobacterial sequences utilized tri-, tetra-, penta-, and hexanucleotide frequency pattern correlations [25]. Four clusters of scaffolds from an initial co-assembly of all metagenomic samples were determined to be of sufficient size for analysis and were confirmed to possess flavobacterial phylogenetic markers. These clusters were assigned as phylogenetic bins (FlavA, FlavG, FlavH, and FlavI) and were assembled, annotated, and screened for duplicate assemblies. Based on the conserved set of genomic functions and single-copy genes, we estimated the degree of completeness for each bin to be 37%–84% and duplication to be 0%–7% (Table 1; Additional file 2: Table S2). Due to the size of some of the bins, the number of commonly used phylogenetic markers was limited. For example, only a single bin (FlavA) had a copy of the 16S rRNA gene, and only two bins had a copy of the 23S rRNA gene. Two phylogenetic markers common to all four bins were DNA primase (DnaG) and ATP-dependent DNA helicase (RecG), and several other markers were present in three of the four bins. The phylogenetic relationship of these bins within the *Flavobacteriaceae* is similar for all of the marker genes; FlavA and FlavH cluster with *Flavobacteria* sp. MS024-2A (Figure 1; Additional file 1: Figure S3A–D), a single cell amplified *Flavobacteria* from the GoMA [26], and are basal to the rest of *Flavobacteriaceae* in four of the five constructed trees. FlavH and FlavI form a more divergent clade, are still closely related to the Flavobacteria from the GoMA, and are always basal to the FlavA, FlavH, and MS024-2A clade. FlavH, which had an estimated degree of duplication of 7%, possessed two copies of RecG that share 77% amino acid identity and is likely a result of the binning method incorporating closely related organisms into a single bin.

**Table 1:** Summary of the identified phylogenetic bins used in this study

**Bin name**	**Total size (bps)**	**Number of scaffolds**	**Percentage (%) complete**	**Percentage (%) duplication**	**Approximate genome size (bp)**
FlavA	2,220,739	19	83.60	0.00	2,656,386
FlavG	703,361	4	36.80	2.01	1,872,889
FlavH	1,453,303	15	57.20	7.04	2,361,871
FlavI	799,420	10	37.92	0.00	2,108,175

### Spatial and temporal differentiation

The GoMA metagenome spans several sites and two seasons, allowing for comparison within and between bins on both temporal and spatial scales. As a whole, flavobacterial sequences are more than twice as abundant during the summer compared to the winter (Table 2). Fluctuations in flavobacterial abundances between seasons, while *Bacteroidetes* abundances remain constant are evidence of dynamic changes in population structure at lower taxonomic levels. Surface chlorophyll-*a* concentrations (and consequently standing biomass) are higher in the GoMA during August (Figure 1). Scaffolds within each bin have similar abundances for each season, adding supporting evidence that these phylogenetic bins represent discreet, cohesive units (Additional file 2: Table S3). However, several outliers are apparent (Additional file 2: Table S3; as determined using [27]). These outlier scaffolds are possibly the result of stochastic variation caused by the random nature of environmental sampling or the biases involved with cloning and Sanger sequencing methodologies. Outliers could also possibly represent seasonally dependent content differences within a bin. Scaffolds considered to be outliers based on seasonal composition were not included in biogeographical analysis but were included for the analysis of gene content variation.

**Table 2:** Data pertaining to the seasonality and spatial heterogeneity of sequences from each bin

	**Percent of reads in the assemblies by season**^**a**^		**Percent abundance of reads by season**		**Percent abundance of reads by site**^**b**^					
**Bin**	**Winter**	**Summer**	**Winter**	**Summer**	**GoMA03**	**GoMA04**	**GoMA06**	**GoMA12**	**GoMA13**	**GoMA14**
FlavA	6.52	93.5	0.04	0.63	0.05	0.04	0.00	1.22	0.33	0.68
FlavG	20.3	79.7	0.04	0.16	0.05	0.04	0.02	0.18	0.16	0.13
FlavH	40.7	59.3	0.10	0.14	0.11	0.09	0.10	0.21	0.11	0.22
FlavI	60.7	39.3	0.14	0.09	0.11	0.15	0.16	0.12	0.07	0.06
TOTAL	-	-	0.32	1.03	0.33	0.32	0.28	1.73	0.67	1.09

^a^Percentage of the total number of sequences that compose all assemblies within each season.

^b^Percent abundance of sequences identified in each bin from corresponding libraries (Number of sequences ÷ Total sequences from library [or season] × 100).

All four bins are present in both summer and winter. FlavA is significantly more abundant in the summer than FlavH or FlavI (*t* test, *p* < 0.002), and conversely, FlavI is significantly more abundant than FlavA or FlavH in the winter (*t* test, *p* < 0.002). FlavA and FlavG are composed of sequences primarily derived from the summer, for which FlavA is almost exclusively present at this time, while FlavG has more presence during the winter. FlavI is made up of primarily winter sequences (abundances in the summer, FlavA > FlavG > FlavH > FlavI). Each of the four environmental genomes was a rare member of the total community; derived from organisms that collectively compose about 1% of the total microbial metagenome.

It is unknown the degree to which *Flavobacteriaceae* share overlapping ecological niches or the extent to which species specialize in substrate degradation or distinct microhabitat conditions. Using the GoMA metagenomic data, we explored the possibility that the organisms represented in each bin may temporally overlap but are spatially separated. Previous work has shown that microbial bacterioplankton community can change over short distances (10 km) [28] and possess the largest difference in community structure at the 6-month temporal scale [29]. Our data support both homogenous mixing across a large spatial-scale and spatial separation, depending on the population. In August, FlavH had near-identical abundance at two different sites (GoMA12 and GoMA14; 0.213% and 0.215%, respectively; *p* = 0.0234, 10,000 permutation *t* test using DAAG package in R), separated by 221 km. FlavA was far more abundant at site GoMA12 and much less abundant at site GoMA13 (1.218% and 0.331%, respectively; *p* < 0.002, 10,000 permutation *t* test DAAG package). Generally, for each season, the most abundant organism for that season is spatially heterogeneous, while the less abundant organisms are more homogeneous (Table 2). The heterogeneous nature of the high-abundance organisms may represent localized blooms related to the current conditions and available substrates, while the low-abundance organisms represent a background population adapted for different conditions and substrates. The data presented here cannot be used to directly explore this hypothesis, though it can be used to look at how organisms competing for limited resources may selectively occupy distinct niches by utilizing a different suite of genomic adaptations.

### Phylogenetic bin functionality

Functionality is difficult to determine due to the incomplete nature of the phylogenetic bins. Parsing bins for genes found in other *Flavobacteriaceae*, illustrates the patchy nature of the metagenomic coverage; however, it does reveal some commonality of genes and functions amongst all four bins. For example, all bins possess genes involved in gliding motility. The bins possess either phosphoenolpyruvate (PEP) carboxylase (FlavH), malic enzyme (FlavG and FlavH), or both (FlavA), key genes that perform anaplerotic carbon fixation, an energy intensive process that may be important for replenishing cellular intermediates in conjunction with proteorhodopsin activity [30] (Additional file 2: Table S4). FlavA does not include an identified proteorhodopsin gene, though it may simply be in a sequencing gap. The other three have green-light adapted proteorhodopsin [31] (Additional file 1: Figure S3B). FlavG, FlavH, and FlavI lack genes related to cobalamin (vitamin B12) metabolism, while FlavA possesses an outer membrane receptor and ATP:cob(I)alamin adenosyltransferase (PduO), which converts vitamin B12 into the coenzyme form [32]. Genes involved with the processing of thiamin (vitamin B1) can be found in FlavA (thiamin-monophosphate kinase and thiamin biosynthesis lipoprotein, ApbE) and FlavG (three copies of ApbE), while none were found in FlavH and FlavI. FlavA, FlavH, and FlavI share superoxide dismutases that have a copper-zinc (Cu-Zn) coenzymatic active site, while FlavG lacks an identified superoxide dismutase. FlavA also possesses a second dismutase with a manganese coenzymatic site.

Further, the genomic content within the phylogenetic bins was searched for putative genes that would potentially confer unique ecophysiological phenotypes within the GoMA Flavobacteria. Only putative coding DNA sequences (CDSs) that lacked sequence similarity with CDSs in the available, fully sequenced *Flavobacteriaceae* (Additional file 2: Table S5) and the GenBank nonredundant (NR) database were explored. Putative CDSs without functional annotation were not included in this analysis. While (conserved) hypothetical genes are likely to provide novel functional mechanisms, it is beyond the scope of this analysis to attempt to determine such functions. Additionally, putative proteins with divergent amino acid sequences were not assessed as it was difficult to determine what appropriate cutoff of sequence dissimilarity should be applied as slight variations in amino acid sequence can alter specificity and substrates, and conversely, high variation can have limited impacts on function [33].

Putative CDSs without sequence similarity to the other *Flavobacteriaceae* and the GenBank NR were identified for each phylogenetic bin, ranging from 16 in FlavI (2.1% of predicted CDSs) to 46 in FlavA (2.1% of predicted CDSs) (Additional file 2: Table S2). Each identified gene was assessed for relevance for this analysis and putative CDSs with necessary cellular functions (e.g., SSU ribosomal protein S16P) were removed. Due to the incomplete nature of these bins, the following identified genes cannot be attributed to a single organism; however, collectively, these putative genes represent genomic content unique to Flavobacteria in the GoMA.

### Novel genes with putative ecophysiological relevance

When the CDSs of FlavA were compared to the other Flavobacteria and the GenBank NR, a number of novel genes were identified that suggests that attachment to particles may be important. Three CDSs identified have possible roles in cell adhesion: a YapH protein (a large protein family that include adhesins), a fat protein possibly involved in cell-cell attachment (UniProtKB ID. Q7UY44), a protein rich in Ca^2+^-binding sites, and a CDS with multiple VCBS domains (PF13517). Further, two CDSs were identified as containing TSP (thrombospondin) type-3 repeats, calcium binding proteins commonly found on the outer membrane [34].

FlavG had three novel CDSs of interest with no similarity to the other Flavobacteria or the GenBank NR: a novel chitinase, an outer membrane lipoprotein that is part of the resistance-nodulation-cell division (RND) efflux system, and calcium-binding protein of the repeats-in-toxin (RTX) family. All three of these genes could potentially be cell surface proteins that interact with or degrade particulate organic matter. Chitinases must act on molecules much larger than can be transported in to the cell [35]. Though RTX proteins have a wide range of functions, one of the more common functions is to act as a surface layer protein, possibly as a peptidase or lipase [36]. These genes suggest interaction with and degradation of particulate organic matter.

Both FlavH and FlavI are smaller bins and have a limited number of novel CDSs. For FlavH, one of the genes without a similar sequence is a lysine exporter (LysE) that has been shown to specifically remove excess lysine from the cellular environment [37]. FlavI genes without sequence similarity in the GenBank NR include a Fe^2+^ siderophore transporter, suggesting diversity in iron transport systems, a gene of unknown function related to a cartilage oligomeric matrix protein, found in humans and some bacteria, and a glycoprotein that contains extracellular and calcium-binding domains [38].

Of particular interest were both peptidases and glycoside hydrolases (GHs). These gene groups have a large diversity of form and function in regards to degrading peptides and large carbohydrates, respectively, and can play key roles in differentiating metabolic potential for organisms and microbial communities.

### Peptidases

Using the estimated degree of completeness and extrapolating, the number of peptidases identified in each bin was within the range commonly seen for flavobacterial genomes (67–149 peptidases) (Table 3). The function of peptidases ranges from the internal cycling of proteins within the cell to interactions with dissolved and particulate organic matter in the cytoplasm, at the cell exterior and or the extracellular environment. Of the identified peptidases, almost half have predicted localization associated with the cytoplasm or cytoplasmic membrane (39%–44%). About half of the peptidases lack a discernable predicted destination (36%–47%). The remaining peptidases could be assigned as either periplasmic (2%–13%) or interacting with the surrounding environment, as either outer membrane-associated or extracellular peptidases (4%–13%).

**Table 3:** The number of peptidases and glycoside hydrolases identified within each bin

**Bin name**	**Total peptidases**	**Cytoplasmic-related peptidases**	**Periplasmic peptidases**	**Cell exterior peptidases**^**a**^	**Unknown peptidases**	**Unique peptidases**^**b**^	**Total glycoside hydrolases**	**Unique gylcoside hydrolases**^**b**^
FlavA	57	25	1	4	27	2	33	3
FlavG	23	9	3	1	10	1	7	1
FlavH	52	23	3	7	19	2	9	0
FlavI	23	9	3	1	10	0	14	1

^a^Cell exterior peptidases include peptidases predicted to be associated with the outer membrane and extracellular.

^b^Unique refers to a lack of significant similarity when searched against the GenBank NR.

All of the bins, except for FlavI, have at least one peptidase without similarity to the Flavobacteria genomes or the GenBank NR database (Table 3). These five peptidases were either assigned a destination to the outer membrane-associated or extracellular (60%) or, if assigned an unknown destination, had a lipoprotein signal, suggesting that all of the unique peptidases were interacting with proteins in the environment. All of these peptidases belong to the clan MA (CL0126) in the Pfam database. This clan consists of 52 families of peptidases, all of which share a two Zn-dependent co-catalytic site within the motif HEXXH. One of the peptidases from FlavA could be assigned more specifically to family Peptidase M28 (PF04389), which preferentially releases basic amino acids from the N-terminal end of peptides [39].

### Unique glycoside hydrolases

The number of identified GHs for the phylogenetic bins was similar to the numbers found in other marine flavobacterial genomes (18–58 GHs) (Table 3). Each bin, except FlavH, had at least one unique GH when compared to the other Flavobacteria genomes and the GenBank NR. Three of the unique GHs were derived from FlavA. The first unique GH contained a domain assigned to GH Family 88 (PF07470), annotated as a rhamnogalacturonide degradation protein, indicating it may degrade pectin polysaccharides [40]. The second was assigned to the GH Family 18 (PF00704), which has a number of possible functions attributed to it, such as chitinases, acetylglucosaminidases, and xylanase inhibitors [40]. The third was annotated as a short-chain dehydrogenase/reductase and was assigned as a GH with a bacterial neuraminidase repeat (BNR). FlavI also contained a unique CDS with putative BNR function. Neuraminidases specifically degrade neuraminic acids, commonly found glycoproteins on cell surfaces [41]. Activity of the putative neuraminidases would require the Flavobacteria populations to be attached to particles with glycoproteins proteins present, further supporting the putative role of Flavobacteria attached to particles, either cellular or detrital in nature. FlavG contained a single unique GH that was annotated as an α-l-fucosidase, which will cleave hexose deoxy sugars on cell surfaces [40].

These genes represent those unique to the GoMA Flavobacteria in relation to the other sequenced *Flavobacteriaceae* and all other microbial genomes in the GenBank NR. Many of the identified putative functions and/or protein domains are indicative of interacting with the extracellular environment. These predicted gene functions support a hypothesis of marine Flavobacteria playing a role in the degradation of organic molecules and being associated with marine aggregates, such as detritus and planktonic protozoa.

### Comparative genomics

**Figure 2:**
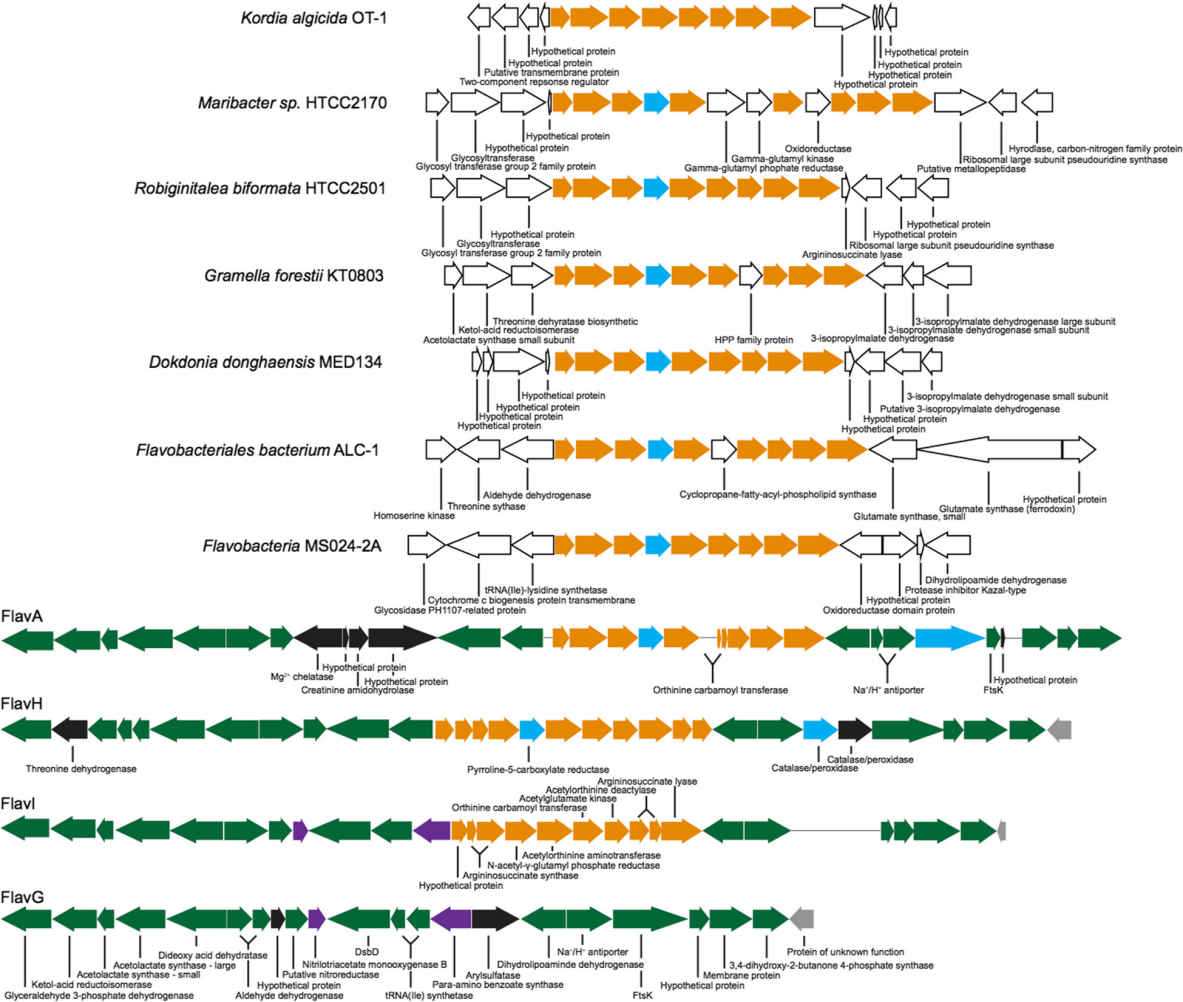
**Gene synteny map of a ~21 kbp homologous region identified using the progressiveMauve aligner.** Annotations were generated using the RAST service. Arrows indicate putative CDS and the direction indicates strand orientation. CDS are color coded to indicate genes common between phylogenetic bins (green = identified in all four bins; black = only present in corresponding bin; purple = identified in FlavI and FlavG; grey = identified in FlavH, FlavI, and FlavG; orange = identified in FlavA, FlavH, and FlavI; blue = identified in FlavA and FlavH).

**Figure 3:**
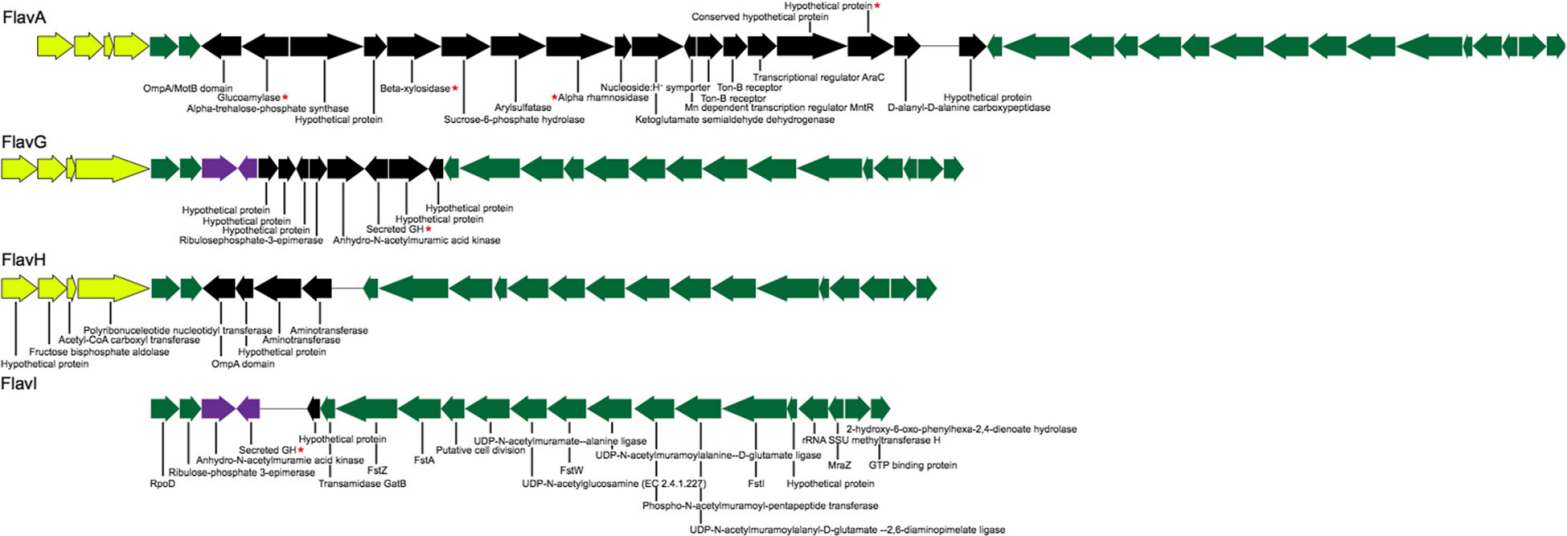
**Gene synteny map of a ~64 kbp homologous region identified using the progressiveMauve aligner.** Annotations were generated using the RAST service. Arrows indicate putative CDS; the direction of which indicates strand orientation. CDS are color coded to indicate genes common between phylogenetic bins (green = identified in all four bins; black = only present in corresponding bin; purple = identified in FlavI and FlavG; yellow = identified in FlavA, FlavG, and FlavH. Red asterisks denote putative CDS with identified glycoside hydrolase domains.

Insight into how genomic diversification occurs can further be explored through examining syntenic genomic regions for evidence of gene addition or deletion. There were only a few syntenic regions between all bins that could be utilized for comparative analysis. Two regions were identified based on nucleotide identity and synteny (Figures 2 and 3). These regions further underscore the high degree of genomic variation that commonly occurs between related organisms and offers insights in to how the phylogenetic relationships may impact genomic content.

Homologous region 1 (HR1) is 15 genes and ~21 kbp in length (Figure 2). Several of the genes appear to have an operon-like nature and pertain to amino acid biosynthesis and other essential cellular functions. Interspaced between the syntenic regions of HR1 reside a number of across-bin variations. The largest variation is the inclusion of genes related to arginine biosynthesis in FlavA, FlavH, and FlavI. Arginine biosynthesis genes are orthologous and syntenic in both FlavA and FlavH; however, both of these bins contain an insertion (relative to FlavI) annotated as pyrroline-5-carboylate reductase. When arginine biosynthesis genes are compared to homologs in the genomes of the *Flavobacteriaceae* used to identify unique Flavobacteria genes in the GoMA (Additional file 2: Table S5), it can be seen that the whole pathway is syntenic for most of the organisms, with exception of *Kordia algicida* OT-1, which, like FlavI, lacks pyrroline-5-carboylate reductase. However, unlike the GoMA phylogenetic bins, the surrounding genomic architecture varies quite markedly, including Flavobacteria sp. MS024-2A. This suggests that the arginine biosynthesis operon is conserved in members of the *Flavobacteriaceae*, though its relative location in each genome can vary and that the lack of the operon in FlavG may be the result of the rearrangement of the entire operon.

Homologous region 2 (HR2) is far more variable in size, with 17 genes anchoring the syntenic region, and a number of gene differences in FlavA, FlavG, and FlavH that increase the length of the region from 31 kb in FlavI to 64 kb in FlavA (Figure 3). The conserved portion of HR2 indicates a role in cell division. The variable region appears to be amenable to gene gain/loss, and many of the genes present in each bin confer potential functions that would be beneficial to the proposed particle-associated lifestyle of marine *Flavobacteriaceae*, such as glycoside hydrolases and TonB-receptors. Interestingly, the genes within the variable region have limited overlap in putative function between the bins. This may indicate that this type of variable region is prone to gene gain/loss, potentially as a mechanism for incorporating horizontally transferred functions into the genome, and like other genomic islands [24], this region is adjacent to a tRNA, specifically tRNA-Arg. The horizontally transferred genes may confer metabolic specialization and diversification, similar to those genomic islands seen in *Prochlorococcus*[23].

### Gene variation

In addition to evaluating differences in genomic content, we analyzed gene variations within the underlying population structure, using the individual reads that assembled into each putative CDS. The large assemblies and binning protocol allows for analysis of *in vivo* variations in putative CDS of an environmental population. Additionally, Sanger sequencing has lower error rates than 454 and Illumina sequencing platforms (San Diego, CA, USA) allowing for higher confidence in the accuracy of individual base pairs. Further, Sanger sequencing is not subject to amplification bias (though cloning biases do apply) allowing for a more accurate interpretation of the abundance of different gene variants. Due to the disparity between sequence depth (~2 Gbp) and the total genomic content of the sample (~2 Tbp, assuming 5 × 10^6^ cells · ml^-1^ and 2 Mbp · genome^-1^), it is possible to treat each sequence read within a scaffold as being derived from an individual microbial cell. As such, analysis of individual reads reveal a more accurate abundance of identified SNPs. Analysis of SNP variations has rarely been performed on metagenomic datasets because of the low coverage of less abundant organisms in complex environmental metagenomes [31] and the lack of coherent phylogenetic groups to pursue concerted effort within a single population. As such, these analyses usually rely on genomes derived from cultured and sequenced closely related strains. As these problems are addressed, such analyses will be even more relevant for understanding variation in the environment, as the gene variations present within microbial populations may confer an unknown degree of niche differentiation. Many of the identified variant differences from this study are synonymous, but the nonsynonymous changes may have significant effects on protein function and activity.

**Figure 4:**
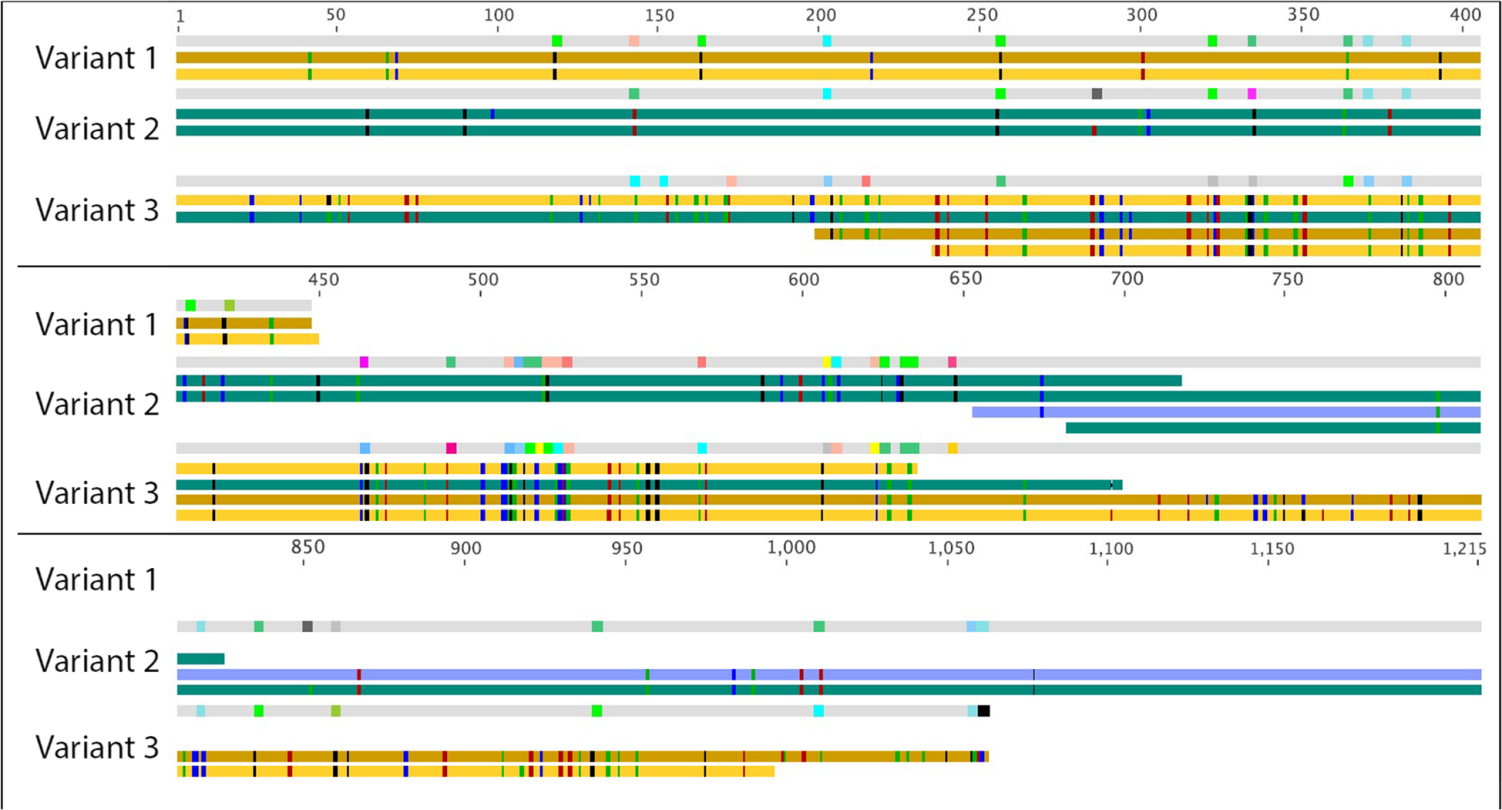
**Diversity of the underlying reads for putative CDS annotated as phospho-N-acetylmuramoyl-pentapeptide transferase.** Each variant has a final protein sequence (grey bars). Below are the nucleotide sequences used to reconstruct the protein. The nucleotide sequences are color coded based on season and sampling location (yellow colors = summer; blue colors = winter; gold = GOM13; yellow = GOM12; blue-green = GOM04; blue = GOM06). Unique sequence reads were aligned to the assembled nucleotide sequence using CLUSTAL W. The colors represent SNP locations in relation to the assembled nucleotide sequence (green = A; blue = C; black = G; red = T). The three protein variants were aligned to each other using CLUSTAL W. Colors along the length of the protein represent amino acid changes.

Putative CDSs were identified with coverage similar to that of a draft genome (>7× coverage) in all of the phylogenetic bins. The 93 CDSs (1.9% of predicted CDS) with sufficient coverage were manually accessed (see the “Methods” section) for SNPs that could be used to differentiate between different gene variants (Table 4). Thirty-three of the CDSs had no SNPs and represented genes for which only one variant was detectable in the environment. A further 16 CDSs had at least one SNP, but variants could not be determined due to a number of factors, such as limited coverage across the length of the gene. For the remaining 44 CDS, 101 different gene variants were generated (Additional file 2: Table S6). One example of a CDS with high coverage is phospho-N-acetylmuramoyl-pentapeptide transferase in FlavG (Figure 4). Three distinct variant tracks that occurred along a 449-bp length of the gene (1,215 bp) were identified through SNP analysis. Variant-1 was the shortest region reconstructed and was composed of two summer clones. Variant-2 and Variant-3 were composed of four clones. All of the clones in Variant-3 were winter clones, while Variant-2 had clones from both seasons. Across the region covered by the three variants there were 47 synonymous and 13 nonsynonymous SNPs in at least one sequence. Of the 102 gene variants, 36 variants were generated that were composed of only summer reads, 23 variants with only winter reads, and 42 variants with reads from both seasons. A majority of the CDSs analyzed in this way (64%) had at least one variant with reads from both seasons. These data indicate that the underlying population structure of the studied *Flavobacteriaceae* has a complex nature, with gene variants present in the population in both the summer and winter competing with seasonally specific variants. Such seasonal variations mask many variables that may be impacting variant presence/absence, such as nutrient availability, water temperature, and grazing pressure. How these variations influence genomic function cannot be parsed from the current information, but the variant can be used to assess *in situ* selective pressure within the population.

**Table 4:** Breakdown of variant data for each bin

**Bin name**	**Number of CDS with >7× coverage**	**Number of CDS without SNPs**	**Number of CDS with multiple variants**	**Number of winter only variants**	**Number of summer only variants**	**Number of variants composed of both seasons**
FlavA	43	24	13	1	22	4
FlavG	24	5	15	4	12	20
FlavH	11	1	9	5	2	14
FlavI	11	3	7	13	0	4

The presence of multiple closely related individuals within each phylogenetic bin allows for a comparison of selection pressures using dN/dS values. dN/dS is calculated based on the number of nonsynonymous substitutions per nonsynonymous site to the number of synonymous substitutions per synonymous site. Differences can be used as an indicator of selective pressure acting on a protein-coding gene. Further, dS can be used to approximate the time since divergence, if there is the assumption of a constant mutation rate across all genes [42]. This procedure is not commonly applied to environmentally derived genomes and datasets [18-20] but has the potential to elucidate further understanding of how selective pressures are manifested in the environment.

**Figure 5:**
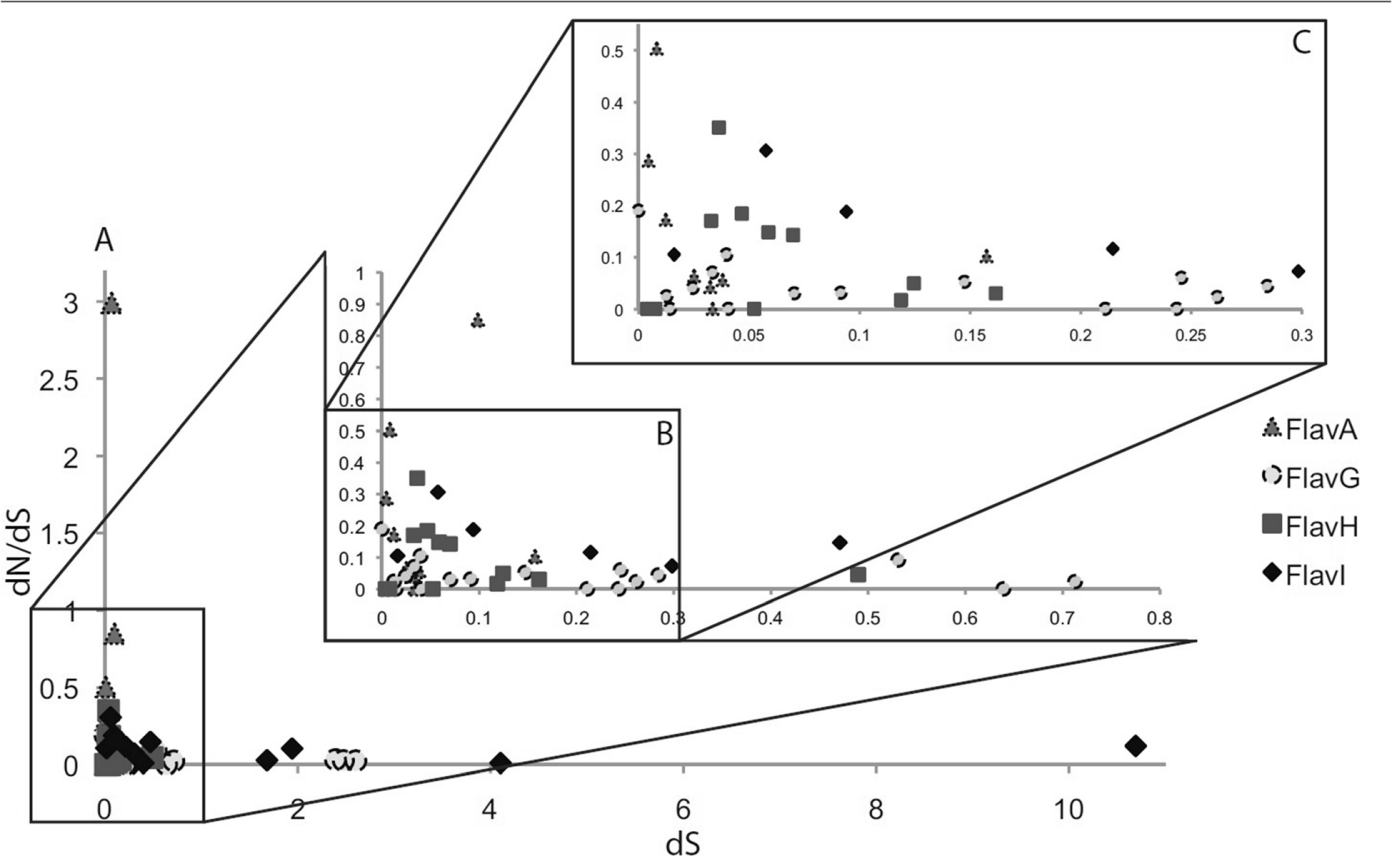
**dN/dS ratio plotted against dS.** dN/dS and dS values were calculated by comparing the identified proteins and nucleotide sequences for each variant pair using the PAL2NAL software and the codeml program within PAML. **(A)** All variants and corresponding dS and dN/dS values. **(B)** Enlargement of the indicated section of **(A)**. **(C)** Enlargement of the indicated section of **(B)**.

Using our data, dN/dS values were calculated for each variant pair to determine the selective pressure on each gene (in the cases with CDS that had more than two variants, pairwise comparisons were made against the variant with the most underlying sequences) (Figure 5). All but one CDS appear to be under purifying selection (dN/dS < 1). The single CDS under positive selection was annotated as a hypothetical protein, 114 bp in length, with no significant similarity in the NR, suggesting it may just be an artifact of the annotation process. Several CDSs from FlavG and FlavH have a dS value > 2.0, a cutoff generally used to classify genes that are saturated in synonymous mutations, such that the estimate of dS is inaccurate [42]. The sharp distinction between these CDSs and most of the other variants (dS < 0.9) may suggest that CDS with dS > 2.0 have a disparate gene history compared with the rest of the genome, potentially the result of horizontal gene transfer and recombination. This technique offers a way of identifying genes that undergo recombination amongst individuals of a population, offering insight into the extent at which recombination occurs *in situ* at sites away from recombination markers, such as transposons and genomic islands. Many of the rest of the CDS have dS values < 0.3, indicating that individuals within each phylogenetic population have a relatively short time since divergence, and low dN/dS value (<0.4), supporting removal of nonsynonymous mutations from the population.

## Conclusions

The analysis of the flavobacterial component of the GoMA metagenome has been used to understand how community and population diversity are separated both spatially and temporally in the planktonic environment. We were able to identify Flavobacteria populations that were abundant in the different seasons and illustrated that the abundant populations exhibited a heterogeneous distribution between the sites compared to the homogeneous distribution of the less abundant organisms. Using comparative genomics, it is possible to identify potentially key differences in the gene content of these flavobacterial populations such as unique iron transporters (FlavI) and specialization in cell attachment (FlavA) and polymer exploitation (FlavG). Further, we were able to see similar gene content over large segments of syntenic regions for all four bins, including observations regarding the variable nature and gene content of the arginine biosynthesis operon and the identification of a genomic island that may play a role in the acquisition of new functions. We were able to start examining the selection pressures on specific genes and could see evidence of recombination between populations. The utility of metagenomics as a tool for microbial ecology will only increase as methods for analyzing large-scale genomic data can be applied to both hypothesis-generating and hypothesis-testing scenarios, such as testing the functional variation of similar microbial population;, examining the presence, abundance, and extent of gene variations in the underlying populations; and exploring the impacts such variations may have on community function.

## Methods

### Metagenome sequencing

As previously described [14], three 200 L water samples were collected from 1.5-m depth from the GoMA in January and August of 2006 (Additional file 2: Table S1), size-fractionated by sequential filtering, immediately frozen in liquid N_2,_ and stored at -80°C. Sequencing of the metagenome has been described previously [14] and follows established protocols [31]. In brief, DNA was extracted from organisms collected in the 0.1–0.8 μm filter range, inserted into medium range insert vectors (3–10 Kbp), and sequenced via paired-end Sanger sequencing. A total of 2,827,702 reads were generated for six samples containing over 2,235 Mbp of sequence data (mean read length 1,008 bp). All sequences were subjected to quality screening using phred [43] and an initial co-assembly of all the sequence data with the Celera assembler at the J. Craig Venter Institute following established protocols [31].

### Metagenomic binning

Single-copy, non-16S small subunit rRNA phylogenetic markers, as identified in [44] from *Flavobacterium psychrophilum* JIP02, were searched against all GOM scaffolds using BLASTN (all values used in BLAST searches can be found in Additional file 2: Table S7). All scaffolds were then grouped using an oligonucleotide frequency calculator. All scaffolds >3,000 bp in length had tri-, tetra-, penta-, and hexanucleotide frequencies determined. Scaffolds were clustered using a hierarchical clustering method. Scaffolds with phylogenetic markers were used to identify all clusters of scaffolds with a Pearson's correlation ≥0.90. Such clusters were considered for further analysis. Clusters with cumulative length at least as long as previous *Flavobacteriales* genomes (>1.9 Mbp) (*n* = 3) were individually re-assembled using the Celera assembler (see supplemental materials and methods for parameters in Additional file 3) and, collectively, all scaffolds generated from these assemblies >5,000 bp were grouped using the oligonucleotide frequency calculator. Only clusters of scaffolds containing >10,000 reads were identified for further assessment. The reads compromising each cluster were re-assembled with the Celera assembler separately. If the estimated percent duplication of single-copy genes (see below) was high, the reads comprising each cluster were further sub-divided until the duplication estimate was <10%. The subdivided clusters were assembled separately using the Celera assembler. All assembled clusters remaining were determined to represent phylogenetic bins (i.e., partial environmental genomes). Putative phylogeny was determined by identifying the 16S SSU rRNA gene. If a bin had a 16S rRNA gene without similarity to the *Flavobacteriaceae*, it was excluded from further analysis.

### Annotation and bin refinement

Phylogenetic bins were annotated using the RAST annotation server [45]. The accuracy of the scaffolds within each bin was further refined using a process similar to [14]. In brief, each putative CDS was assigned a phylogenetic identity based on a top hit using BLASTP to query the NCBI GenBank NR database [46]. If the percent of CDS on a scaffold with a top hit to the phylum *Bacteroidetes* was <60%, the scaffold was removed from the bin. Duplicated scaffolds were identified using a BLASTP search of all putative CDS within a bin against the CDS of *Flavobacteriales* sp*.* ALC-1. If the smaller of the two scaffolds had ≥85% similar genes to a longer scaffold, the shorter scaffold was removed from the bin. All duplicated scaffolds were confirmed by progressiveMauve alignments [47].

### Estimation of bin completeness and duplication

Estimation of bin completeness and duplication was adapted from [10]. Seven marine-related *Flavobacteriaceae* genomes (Additional file 2: Table S5) were identified to develop a “pangenome” related to the bins. Using HMMER V3.0 [48] (E-value ≤ 1E-5), all CDS from each of the seven genomes within the pangenome were searched against the TIGRFAM database V12.0 [49]. For each TIGRFAM match common in all genomes, a minimum shared number for each individual TIGRFAM match was determined to be the core genome. The core genome consisted of 665 unique TIGRFAM models, and 799 total occurrences. Percent completeness for each bin was determined using the equation:

$$\text{est}.\text{\%}\;\text{complete}=\frac{\text{No}.\;\mathrm{of}\;\text{identified}\;\text{core}\;\text{genes}}{\mathrm{No}.\;\mathrm{of}\;\text{expected}\;\text{core}\;\text{genes}}\times 100$$

The same pangenome was used to determine the minimum number of single-copy genes via BLASTP, and 199 conserved single-copy genes (CSCGs) were identified. Percent duplication within a bin was determine via a BLASTP search of the CSCGs against all putative CDS and determined using the equation:

$$\text{est}.\%\;\text{duplication}=\frac{\text{No}.\;\text{of}\;\text{duplicate}\;\text{genes}}{\text{No}.\;\text{of}\;\text{CSCGs}}\times 100$$

### Identification of catalytic domains

Catalytic domains for GHs and carbohydrate-binding modules (CBMs) identified from [10] and peptidases were identified by comparing the MEROPS database [50] of catalytic peptidase units against the PFAM V26.0 to identify the best PFAM model for each peptidase unit. These lists were used to generate libraries from PFAM V26.0. Each library was used to search the putative CDS from each bin using HMMER. The peptidases of each bin were compared via BLASTP against each other, the pangenome, and the GenBank NR. The predicted destinations for the peptidases were determined using PSORTb (v3.0.2) [51], signal peptides determined by SignalP (v4.1) [52], and lipoprotein signals determined by LipoP (v1.0) [53].

### Population analysis of variable sites

Using Geneious V5.6.2 (Biomatters; http://www.geneious.com), the underlying sequences for each scaffold were trimmed and mapped to the respective scaffolds (see supplemental materials and methods for settings in Additional file 3). SNPs were identified and counted for sites with ≥4× read coverage, where at least two of the reads contained a mutation at that site. Alignments were performed using CLUSTAL W [54] and checked manually for bases with low quality scores (cutoff = 30). Low quality base pairs without supporting reads were corrected to reflect the consensus base at these sites. Putative CDS of interest were identified with coverage >7×. Using the convention of 8× coverage as the cutoff used to demarcate draft level genomes using Sanger sequences, a 7× cutoff was selected to increase the number of analyzed CDS, while preventing the analysis of CDS without suitable coverage for the analysis. Each CDS was manually checked for different variants based on SNP patterns. For CDS with more than two variants, pairwise calculations were made using the variant with the most supporting reads as the reference. If two variants had the same number of supporting reads, the reference was determined to be the sequence with the highest nucleotide similarity to the consensus. Amino acid alignments were performed using CLUSTAL W. PAL2NAL (V14) [55] was used to align the corresponding nucleotide sequences. Codon table 11 (bacterial, archaeal, and plant plastid) was used for translations. The “Remove gaps, inframe stop codons” setting was turned on. dS, dN, and dN/dS values were computed in PAL2NAL using the codeml program within PAML [56]. The dS values are a sufficient indicator of time since divergence, with lower values suggesting less time since divergence.

### Bin functionality variation

Putative CDS from a single bin were compared via BLASTP to the other bins, the pangenome, and the GenBank NR to identify unique genes. By concentrating on the CDSs without similarity in other *Flavobacteriaceae* or the GenBank NR protein database, it is possible to highlight genes that may have an impact on ecophysiology. The list of putative genes of interest was reduced by removing CDS with duplicate annotation genes and manually curated for genes with potential ecophysiological impacts. progressiveMauve was used to compare the scaffolds of the four phylogenetic bins and identify large regions of synteny and homology.

### Phylogenetic and proteorhodopsin marker trees

Putative proteorhodopsin nucleotide sequences were identified for each bin and aligned with CLUSTAL W to sequences obtained from GenBank. The list of phylogenetic markers presented in Santos and Ochman [44] was used to identify putative marker sequences in each bin. Marker genes present in at least three of the four bins were selected (DNA primase (DnaG), ATP-dependent DNA helicase (RecG), DNA polymerase III, alpha subunit (DnaE), ATP-dependent DNA helicase (RecG), translation elongation factor (EF-G) (FusA), and DNA-directed RNA polymerase subunit beta’ (RpoC)) and aligned with CLUSTAL W to sequences obtained from IMG Genomes [57]. It should be noted that RpoC is not as robust of a phylogenetic marker as the corresponding subunit beta (RpoB). Alignments were trimmed, concatenated in the case of the DnaG-FusA, DnaG-DnaE-RecG, and DnaG-RpoB trees, and a maximum likelihood tree was constructed using PHYML with 1,000 bootstraps [58] (see supplemental materials and methods in Additional file 3).

#### Sequence data

Raw reads can be found in the NCBI GenBank Trace Archive (TA) ID No. 2307942905–2310786347. Scaffolds of putative *Flavobacteria* will be deposited after review and acceptance.

## Abbreviations

GoMA: Gulf of Maine; CDS: Coding DNA sequence; CSCG: Conserved single-copy gene; NR: Nonredundant; TSP: Thrombospondin; RTX: Repeats-in-toxin; GH: Glycoside hydrolases; BNR: Bacterial neuraminidase repeat.

## Competing interests

The authors declare that they have no competing interests.

## Authors’ contributions

BJT and JFH designed the experiment. BJT performed the analysis and wrote the manuscript. RS developed the script package used to construct phylogenetic bins based on tetranucleotide frequencies. KBH and JFH received funding and designed the scope of sampling efforts within the GoMA. BJT, RS, KBH, and JFH performed revisions on the manuscript. All authors read and approved the final manuscript.

## Supplementary Material

Additional file 1 **Supplemental figures.** This file provides additional figures as described in the manuscript. Click here for file

Additional file 2 **Supplemental tables.** This file provides additional tables as described in the manuscript. Click here for file

Additional file 3 **Supplemental materials and methods.** This file provides the parameters used for selecting 16S rRNA gene fragments, the Celera Assembler, the Geneious Assembler, Geneious SNP detection, and PHYML tree construction. Click here for file
