# Supplementary material for: Comparative genomics of planktonic Flavobacteriaceae from the Gulf of Maine using metagenomic data
Source: Microbiome. 2014 Sep 5;2:34. doi: 10.1186/2049-2618-2-34 (PMC4164334; doi:10.1186/2049-2618-2-34)
Supplement: Additional file 1 — Supplemental figures. This file provides additional figures as described in the manuscript. [file 2049-2618-2-34-S1.pdf]

Supplemental Figure Legends.

SI Figure 1. MODIS satellite data showing the surface chlorophyll-*a* levels in pre-bloom conditions, averaged over the January (winter) and August (summer) of 2006. The locations of the sampling sites are noted.

SI Figure 2. Number of identified 16S rRNA gene fragments from the Gulf of Maine metagenome from each sampling season for several phylum level groups in the *Bacteria* and *Archaea*, and the class level groups within the *Proteobacteria* (plotted on a different scale). Numbers above each bar indicate the percent of the total sequences each bar represents for each season.

SI Figure 3. Maximum likelihood tree generated using PHYML (bootstrap: 1,000) of: A) DNA helicase (RecG), B) DnaG-FusA, C) DnaG-DnaE-RecG, D) DnaG-RpoC, and E) proteorhodopsin. These genes were identified in the four phylogenetic bins and other members of the Class *Flavobacteriaceae*. Only bootstrap values greater than 50% are shown.

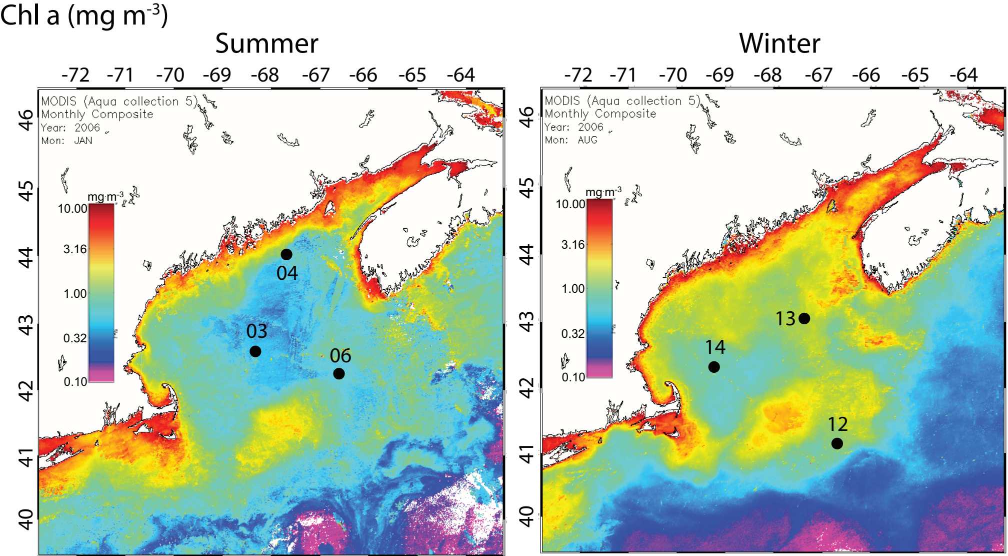

Chl a (mg m-3)
Summer
Winter
-72
-71
-70
-69
-68
-67
-66
-65
-64
46
45
44
43
42
41
40
MODIS (Aqua collection 5)
Monthly Composite
Year: 2006
Mon: JAN
Mon: AUG
mg·m-3
10.00
3.16
1.00
0.32
0.10
04
03
06
13
14
12

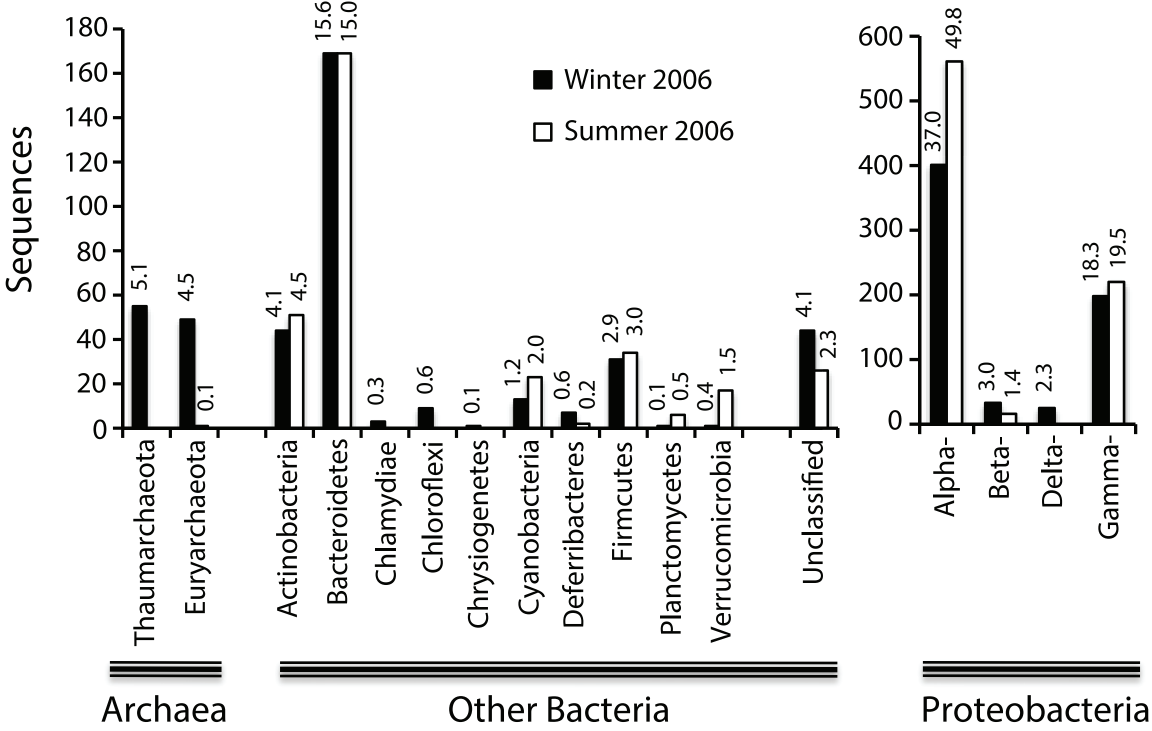

Sequences
Winter 2006
Summer 2006
180
160
140
120
100
80
60
40
20
0
600
500
400
300
200
100
0
5.1
4.5
0.1
4.1
4.5
15.6
15.0
0.3
0.6
0.1
1.2
2.0
0.6
0.2
2.9
3.0
0.1
0.5
0.4
1.5
4.1
2.3
37.0
49.8
3.0
1.4
2.3
18.3
19.5
Thaumarchaeota
Euryarchaeota
Actinobacteria
Bacteroidetes
Chlamydiae
Chloroflexi
Chrysiogenetes
Cyanobacteria
Deferribacteres
Firmcutes
Planctomycetes
Verrucomicrobia
Unclassified
Alpha-
Beta-
Delta-
Gamma-
Archaea
Other Bacteria
Proteobacteria

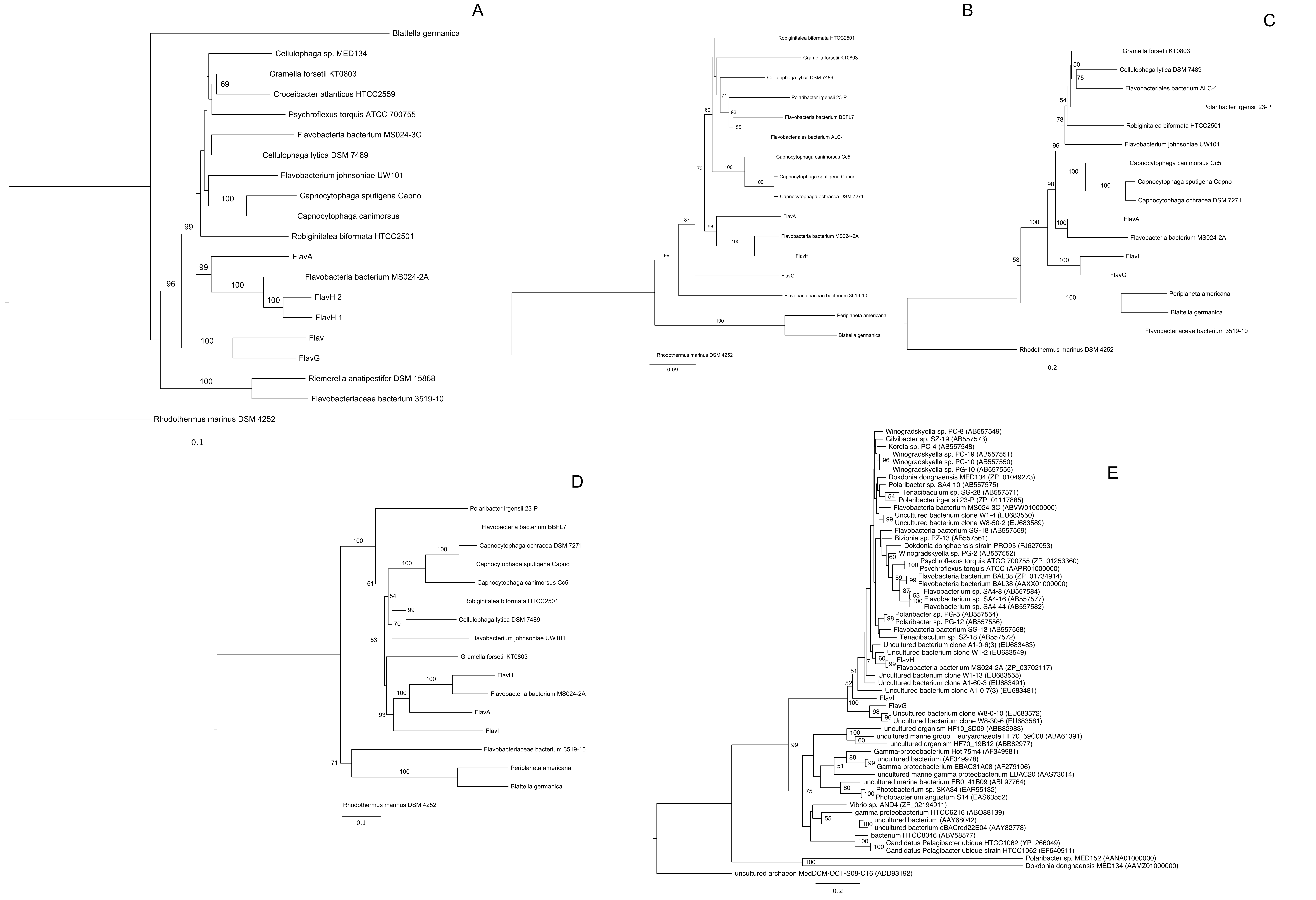
A
Blattella germanica
Cellulophaga sp. MED134
Gramella forsetii KT0803
Croceibacter atlanticus HTCC2559
Psychroflexus torquis ATCC 700755
Flavobacteria bacterium MS024-3C
Cellulophaga lytica DSM 7489
Flavobacterium johnsoniae UW101
Capnocytophaga sputigena Capno
Capnocytophaga canimorsus
Robiginitalea biformata HTCC2501
FlavA
Flavobacteria bacterium MS024-2A
FlavH 2
FlavH 1
FlavI
FlavG
Riemerella anatipestifer DSM 15868
Flavobacteriaceae bacterium 3519-10
Rhodothermus marinus DSM 4252
0.1
B
Robiginitalea biformata HTCC2501
Gramella forsetii KT0803
Cellulophaga lytica DSM 7489
Polaribacter irgensii 23-P
Flavobacteria bacterium BBFL7
Flavobacteriales bacterium ALC-1
Capnocytophaga canimorsus Cc5
Capnocytophaga sputigena Capno
Capnocytophaga ochracea DSM 7271
FlavA
Flavobacteria bacterium MS024-2A
FlavH
FlavG
Flavobacteriaceae bacterium 3519-10
Periplaneta americana
Blattella germanica
Rhodothermus marinus DSM 4252
0.09
C
Gramella forsetii KT0803
Cellulophaga lytica DSM 7489
Flavobacteriales bacterium ALC-1
Polaribacter irgensii 23-P
Robiginitalea biformata HTCC2501
Flavobacterium johnsoniae UW101
Capnocytophaga canimorsus Cc5
Capnocytophaga sputigena Capno
Capnocytophaga ochracea DSM 7271
FlavA
Flavobacteria bacterium MS024-2A
FlavI
FlavG
Periplaneta americana
Blattella germanica
Flavobacteriaceae bacterium 3519-10
Rhodothermus marinus DSM 4252
0.2
D
Polaribacter irgensii 23-P
Flavobacteria bacterium BBFL7
Capnocytophaga ochracea DSM 7271
Capnocytophaga sputigena Capno
Capnocytophaga canimorsus Cc5
Robiginitalea biformata HTCC2501
Cellulophaga lytica DSM 7489
Flavobacterium johnsoniae UW101
Gramella forsetii KT0803
FlavH
Flavobacteria bacterium MS024-2A
FlavA
FlavI
Flavobacteriaceae bacterium 3519-10
Periplaneta americana
Blattella germanica
Rhodothermus marinus DSM 4252
0.1
E
Winogradskyella sp. PC-8 (AB557549)
Gilvibacter sp. SZ-19 (AB557573)
Kordia sp. PC-4 (AB557548)
Winogradskyella sp. PC-19 (AB557551)
Winogradskyella sp. PC-10 (AB557550)
Winogradskyella sp. PG-10 (AB557555)
Dokdonia donghaensis MED134 (ZP_01049273)
Polaribacter sp. SA4-10 (AB557575)
Tenacibaculum sp. SG-28 (AB557571)
Polaribacter irgensii 23-P (ZP_01117885)
Flavobacteria bacterium MS024-3C (ABVW01000000)
Uncultured bacterium clone W1-4 (EU683550)
Uncultured bacterium clone W8-50-2 (EU683589)
Flavobacteria bacterium SG-18 (AB557569)
Bizionia sp. PZ-13 (AB557561)
Dokdonia donghaensis strain PRO95 (FJ627053)
Winogradskyella sp. PG-2 (AB557552)
Psychroflexus torquis ATCC 700755 (ZP_01253360)
Psychroflexus torquis ATCC (AAPR01000000)
Flavobacteria bacterium BAL38 (ZP_01734914)
Flavobacteria bacterium BAL38 (AAXX01000000)
Flavobacterium sp. SA4-8 (AB557584)
Flavobacterium sp. SA4-16 (AB557577)
Flavobacterium sp. SA4-44 (AB557582)
Polaribacter sp. PG-5 (AB557554)
Polaribacter sp. PG-12 (AB557556)
Flavobacteria bacterium SG-13 (AB557568)
Tenacibaculum sp. SZ-18 (AB557572)
Uncultured bacterium clone A1-0-6(3) (EU683483)
Uncultured bacterium clone W1-2 (EU683549)
FlavH
Flavobacteria bacterium MS024-2A (ZP_03702117)
Uncultured bacterium clone W1-13 (EU683555)
Uncultured bacterium clone A1-60-3 (EU683491)
Uncultured bacterium clone A1-0-7(3) (EU683481)
FlavI
FlavG
Uncultured bacterium clone W8-0-10 (EU683572)
Uncultured bacterium clone W8-30-6 (EU683581)
uncultured organism HF10_3D09 (ABB82983)
uncultured marine group II euryarchaeote HF70_59C08 (ABA61391)
uncultured organism HF70_19B12 (ABB82977)
Gamma-proteobacterium Hot 75m4 (AF349981)
uncultured bacterium (AF349978)
Gamma-proteobacterium EBAC31A08 (AF279106)
uncultured marine gamma proteobacterium EBAC20 (AAS73014)
uncultured marine bacterium EB0_41B09 (ABL97764)
Photobacterium sp. SKA34 (EAR55132)
Photobacterium angustum S14 (EAS63552)
Vibrio sp. AND4 (ZP_02194911)
gamma proteobacterium HTCC6216 (ABO88139)
uncultured bacterium (AAY68042)
uncultured bacterium eBACred22E04 (AAY82778)
bacterium HTCC8046 (ABV58577)
Candidatus Pelagibacter ubique HTCC1062 (YP_266049)
Candidatus Pelagibacter ubique strain HTCC1062 (EF640911)
Polaribacter sp. MED152 (AANA01000000)
Dokdonia donghaensis MED134 (AAMZ01000000)
uncultured archaeon MedDCM-OCT-S08-C16 (ADD93192)
0.2
