# Supplementary material for: Comparative genomics of planktonic Flavobacteriaceae from the Gulf of Maine using metagenomic data
Source: Microbiome. 2014 Sep 5;2:34. doi: 10.1186/2049-2618-2-34 (PMC4164334; doi:10.1186/2049-2618-2-34)
Supplement: Additional file 2 — Supplemental tables. This file provides additional tables as described in the manuscript. [file 2049-2618-2-34-S2.pdf]

Supplemental Table Legends.

SI Table 1. Site summary data for samples collected from the Gulf of Maine in 2006.

SI Table 2. Data pertaining to assembly efficiency, the number of predicted coding sequences, and various values for the comparison of identified peptidases and glycoside hydrolases, including the number of unique genes after 1) bin-to-bin comparisons, 2) comparison to the pangenome, & 3) comparison to NR.

SI Table 3. Data pertaining to the seasonality and spatial heterogeneity of sequences from each scaffold within each bin. "By season" is the percentage (%) of the total number of sequences that compose all assemblies within each scaffold by season. "Perc. Abundance of Reads by Season and Sampling Location" is the percent (%) abundance of sequences identified in each bin from corresponding libraries or season. (No. of sequences from bin ÷ total sequences from library [or season]) * denote scaffolds identified as outliers using Iglewicz and Hoaglin (1993).

SI Table 4. Functions of interest used to examine features common in marine microbes and *Flavobacteria*. + denotes the presence of genes representing that function. – represents processes lacking identified genes.

SI Table 5. The seven genomes used in the pangenome.

SI Table 6. The genes identified in each bin with discernable variants used in the dN/dS analysis. The name "hypothetical protein" is used multiple times and denotes the annotation of the CDS and not a unique identifier.

SI Table 7. Values used as cutoffs for the various BLAST analyses, including a brief description to link with the Methods and Material of the main manuscript.

Table S1. Site summary data for samples collected from the Gulf of Maine in 2006.

		Site location					
Site Name	Date Sampled	Latitude	Longitude	Temp. °C	Salinity, ppt	Number of sequences	Insert Size Range (kb)
GoMA03	25-Jan-06	42° 46' 9" N	68° 40' 8" W	6.4	33.0	453,805	3-5 / 2-3
GoMA04	27-Jan-06	44° 07' 5" N	67° 58' 3" W	5.1	32.2	957,737	4-6
GoMA06	30-Jan-06	41° 28' 7" N	69° 6' 0" W	6.2	32.6	10,040	4-6
GoMA12	25-Aug-06	41° 08' 6" N	66° 53' 3" W	17.4	32.0	470,591	6-8
GoMA13	28-Aug-06	43° 23' 3" N	67° 41' 9" W	16.6	32.0	925,793	6-8 / 8-10
GoMA14	29-Aug-06	42° 21' 6" N	69° 23' 8" W	19.6	31.3	9,728	6-8

Table S2. Data pertaining to assembly efficiency, the number of predicted coding sequences, and various values for the comparison of identified peptidases and glycoside hydrolases, including the number of unique genes after 1) bin-to-bin comparisons, 2) comparison to the pangenome, & 3) comparison to NR.

Bin Name	N50	No. of reads in bin	Annotated Putative CDS	No. of peptidases	No. of Glycoside Hyrdrolases	No. of unique peptidase - bin-to-bin	No. of unique peptidase - including pangenome	No. of unique GH - bin-to-bin
FlavA	133,660	9,910	2,168	57	33	24	5	28
FlavG	129,191	3,452	747	22	7	7	3	4
FlavH	174,151	5,832	1,283	51	8	22	2	3
FlavI	93,775	3,434	772	22	13	4	0	13

Table S3. Data pertaining to the seasonality and spatial heterogeneity of sequences from each scaffold within each bin. “By season” represents a breakdown of the total number of sequences that compose all assemblies within each bin and from the season they were derived. “Perc. Abundance of Reads by Season and Sampling Location” is the percent abundance of sequences identified in each bin from corresponding libraries. (No. of sequences from bin ÷ total sequences from library [or season]). * denote scaffolds identified as outliers using ref. 24.

		By season		Percent (%) Abundance of Reads by Season and Sampling Location							
Bin	Scaffold ID	Winter	Summer	Winter	Summer	GOM03	GOM04	GOM06	GOM12	GOM13	GOM14
FlavA	scf6364	8.129	91.87	0.003	0.035	0.004	0.002	0.000	0.066	0.020	0.000
	scf6365	7.967	92.03	0.006	0.070	0.006	0.006	0.000	0.130	0.040	0.082
	scf6366	5.248	94.75	0.000	0.017	0.000	0.001	0.000	0.032	0.010	0.010
	scf6368	5.71	94.28	0.001	0.027	0.001	0.001	0.000	0.055	0.014	0.000
	scf6369	6.539	93.46	0.005	0.074	0.006	0.004	0.000	0.145	0.038	0.061
	scf6370	4.198	95.8	0.001	0.044	0.002	0.001	0.000	0.086	0.023	0.071
	scf6371	6.55	93.44	0.001	0.027	0.002	0.001	0.000	0.054	0.013	0.020
	scf6372	10.26	89.73	0.000	0.008	0.000	0.001	0.000	0.017	0.004	0.020
	scf6374	7.07	92.92	0.000	0.012	0.001	0.000	0.000	0.025	0.006	0.000
	scf6375	7.465	92.53	0.001	0.023	0.001	0.001	0.000	0.041	0.014	0.020
	scf6376	4.427	95.57	0.001	0.030	0.001	0.001	0.000	0.054	0.017	0.082
	scf6377	6.032	93.96	0.002	0.035	0.001	0.002	0.000	0.074	0.014	0.061
	scf6378	4.907	95.09	0.000	0.008	0.001	0.000	0.000	0.015	0.004	0.000
	scf6380	6.125	93.87	0.001	0.020	0.002	0.000	0.000	0.037	0.011	0.000
	scf6381	6.673	93.32	0.001	0.023	0.002	0.001	0.000	0.048	0.010	0.092
	scf6382	8.518	91.48	0.003	0.033	0.004	0.002	0.000	0.066	0.017	0.061
	scf6383	7.257	92.74	0.001	0.022	0.002	0.001	0.000	0.042	0.012	0.041
	scf6384	6.076	93.92	0.006	0.105	0.009	0.005	0.000	0.209	0.053	0.051
	scf6385	3.502	96.49	0.000	0.007	0.000	0.000	0.000	0.016	0.003	0.000
FlavG	scf5919	22.29	77.7	0.028	0.099	0.031	0.027	0.019	0.101	0.099	0.051
	scf5920	17.02	82.97	0.005	0.025	0.007	0.004	0.000	0.030	0.022	0.061
	scf5921	16.74	83.25	0.007	0.037	0.008	0.007	0.000	0.043	0.034	0.020
	scf5923*	58.53	41.46	0.019	0.013	0.015	0.020	0.059	0.028	0.006	0.000
FlavH	scf1523	43.53	56.46	0.006	0.009	0.005	0.007	0.000	0.013	0.006	0.020
	scf1524	43.03	56.96	0.014	0.019	0.012	0.015	0.019	0.028	0.014	0.000
	scf1525	39.43	60.56	0.003	0.005	0.005	0.003	0.000	0.007	0.004	0.000
	scf1526	35.91	64.08	0.001	0.002	0.001	0.001	0.019	0.001	0.002	0.010
	scf1527	50.06	49.93	0.005	0.005	0.006	0.004	0.000	0.006	0.004	0.000
	scf1528	56.87	43.12	0.002	0.001	0.003	0.001	0.000	0.001	0.002	0.000
	scf1530	30.33	69.66	0.004	0.009	0.004	0.003	0.000	0.014	0.006	0.020
	scf1531	40.11	59.88	0.022	0.033	0.025	0.021	0.019	0.053	0.022	0.082
	scf1532	36.7	63.29	0.002	0.004	0.002	0.002	0.000	0.007	0.002	0.000
	scf1534*	97.01	2.982	0.060	0.001	0.087	0.047	0.000	0.000	0.002	0.000
	scf1535*	96.49	3.509	0.019	0.000	0.031	0.014	0.000	0.000	0.000	0.000
	scf1538	39.22	60.77	0.020	0.031	0.025	0.018	0.019	0.045	0.024	0.061
	scf1539	40.07	59.92	0.011	0.017	0.015	0.009	0.019	0.025	0.013	0.000
	scf1541*	94.86	5.135	0.060	0.003	0.092	0.045	0.019	0.002	0.003	0.000
	scf1542	45.49	54.5	0.003	0.004	0.004	0.003	0.000	0.007	0.002	0.020
FlavI	scf6040	19.74	80.25	0.010	0.042	0.011	0.009	0.039	0.046	0.040	0.061
	scf6043	75.79	24.2	0.009	0.002	0.006	0.010	0.000	0.004	0.002	0.000
	scf6044	78.67	21.32	0.006	0.001	0.001	0.008	0.039	0.003	0.001	0.000
	scf6045	58.46	41.53	0.007	0.005	0.009	0.007	0.000	0.008	0.004	0.000
	scf6046	50.89	49.1	0.013	0.013	0.011	0.014	0.000	0.026	0.006	0.000
	scf6047	98.35	1.648	0.025	0.000	0.034	0.021	0.000	0.000	0.000	0.000
	scf6049	48.52	51.47	0.009	0.010	0.008	0.010	0.000	0.020	0.005	0.000
	scf6050	59.36	40.63	0.004	0.003	0.003	0.004	0.019	0.005	0.001	0.000
	scf6053	86.75	13.24	0.023	0.003	0.012	0.028	0.019	0.003	0.003	0.000
	scf6054	84.63	15.36	0.025	0.004	0.012	0.031	0.039	0.003	0.005	0.000

Table S4. Functions of interest used to examine features common in marine microbes and *Flavobacteria.*

Gene/Function of Interest	FlavA	FlavG	FlavH	FlavI
Gliding motility	GldFGHJDCBI	GldJBCGFI	GldJB	GldJCB, GldJ
Anaplerotic	+	+	+	+
Vitamin B12	+	-	-	-
Vitamin B1	+	-	-	+
Proteorhodopsin	-	+	+	+
Superoxide dismutase	Mn & Cu-Zn	Cu-Zn	Cu-Zn	-

"+" denotes the presence of genes representing that function.
"–" represents processes lacking identified genes.

Table S5. The seven genomes used in the pangenome.

Organisms in pangenome	Reference
Maribacter sp. HTCC2170	Oh HM, Kang I, Yang SJ, Jang Y, Vergin KL, *et al* (2011)
Kordia algicida OT-1	Lee HS, Kang SG, Kwon KK, Lee JH, Kim SJ (2011)
Gramella forsetii KT0803	Bauer M, Kube M, Teeling H, Richter M, Lombardot T, *et al* (2006)
Flavobacteriales sp. ALC-1	Publicly available. GOLD ID in IMG Database Gi01424
Flavobacteria MS024-2A	Woyke T, Xie G, Copeland A, Gonzalez J, Han C, *et al* (2009)
Robiginitalea biformata HTCC2501	Oh HM, Giovannoni SJ, Lee K, Ferriera S, Johnson J, Cho JC (2009)
Dokdonia donghaensis MED134	Gonzalez JM, Pinhassi J, Fernandez-Gomez B, Coll-Llado M, Gonzalez-Velaquez M, *et al* (2011)

Table S6. Genes identified with multiple variant tracks used in the dN/dS analysis.

Name/Bin	Scaffold ID	No. of Tracks	Gene Length	Shortest Variant Length	dN	dS	dN/dS	No. summer reads	No. winter reads
FlavH									
**Translation elongations Factor p**	1531	3	567	501					
Track1								2	1
Track2					0.005	0.1616	0.0307	0	2
Track3					0.01	0.0699	0.143	2	0
**SSU ribosomal protein S4p**	1538	3	606	603					
Track1								3	1
Track2					0.0063	0.1245	0.0502	1	1
Track3					0.0021	0.1187	0.0177	2	2
**Protein-L-isoaspartate O-methyltransferase**	1534	2	642	642					
Track1					0.0001	0.0523	0.001	0	3
Track2								0	2
**Hypothetical-1**	1541	2	1062	1005					
Track1					0	0.0039	0.001	1	2
Track2								0	5
**Hypothetical-2**	1538	2	426	426					
Track1					0	0.0076	0.001	1	1
Track2								3	0
**Acetate permease**	1538	2	471	471					
Track1					0.0056	0.0328	0.1705	3	1
Track2								1	1
**Phosphoribosylaminoimidazole-succinocarboxamide synthase**	1531	2	936	711					
Track1					0.0087	0.0587	0.1484	1	1
Track2								1	1
**Aminotransferase, class III**	1538	3	1425	981					
Track1					0.0089	0.0467	0.1845	1	1
Track2								2	1
Track3					0.0128	0.0363	0.3511	1	1
**Sulfatase modifying factor 1 precursor**	1531	2	1065	954					
Track1					0.0225	0.4897	0.0459	0	3
Track2								2	1
FlavI									
**Hypothetical-1**	6044	3	117	117					
Track1								2	2
Track2					0.0694	0.4709	0.1473	0	3
Track3					0.202	1.9411	0.1041	0	2
**Oxidoreductase**	6040	2	1386	1386					
Track1					0.0017	0.0161	0.1056	3	0
Track2								3	2
**Hypothetical-2**	6044	3	168	168					
Track1								1	2
Track2					0.0219	0.2984	0.0735	0	2
Track3					0.0431	4.1031	0.0105	0	2
**Hypothetical-3**	6054	3	408	408					
Track1					0.0177	0.0576	0.3067	0	4
Track2								0	2
**GCN5-related N-acetyltransferase**	6044	2	489	489					
Track1					0.025	0.2145	0.1164	0	3
Track2								0	3
**Thiol peroxidase**	6043	3	450	285					
Track1					0.0177	0.094	0.1883	0	2
Track2								0	3
Track3					0.0478	1.6802	0.0284	1	1
**Cell division protein FtsH**	6054	3	1947	936					
Track1								0	3
Track2					1.2932	10.6917	0.121	0	2
Track3					0.004	0.3968	0.0101	0	2
FlavA									
**Methionyl-tRNA formyltransferase**	6365	2	945	945					
Track1					0.0033	0	99	2	0
Track2								0	1
**Hypothetical-1**	6382	2	114	114					
Track1					0.2055	0.0687	2.9913	3	0
Track2								2	1
**Hypothetical-2**	6376	3	162	162					
Track1					0.0836	0.0985	0.8493	4	0
Track2								3	0
Track3					0.0095	0.0001	99	4	0
**Transcriptional Regulator**	6376	2	1083	1083					
Track1					0.0037	0	99	2	0
Track2								2	1
**Twin-arginine translocation protein**	6382	2	195	195					
Track1					0.0162	0.0082	0.5035	4	0
Track2								3	0
**1-acyl-sn-glycerol-3-phosphate acyltransferase**	6382	2	615	615					
Track1					0.0022	0.038	0.0566	2	0
Track2								3	0
**Membrane protein**	6384	2	561	561					
Track1					0.0021	0.0123	0.1727	3	0
Track2								3	0
**Hypothetical-3**	6376	2	867	843					
Track1					0.0162	0.1574	0.1028	2	0
Track2								4	0
**Hypothetical-4**	6376	2	123	123					
Track1					0	0.0335	0.001	3	1
Track2								2	0
**Inosose isomerase**	6384	2	1053	987					
Track1					0.0014	0.0324	0.0431	3	0

Track2								4	0
**Phosphoglycerate kinase**	6382	2	1188	879					
Track1					0.0016	0.0251	0.0647	4	0
Track2								1	2
**Iron-sulfur Cluster Assembly ATPase protein**	6384	2	747	747					
Track1					0	0.0041	0.001	3	0
Track2								3	0
**Gluconolactonase**	6382	2	1011	1011					
Track1					0.0013	0.0045	0.2869	4	0
Track2								5	0
FlavG									
**NG,NG-dimethylarginine dimethylaminohydrolase**	5919	3	909	723					
Track1					0.0032	0.0126	0.0252	2	1
Track2					0	0.0142	0.001	2	0
Track3								2	1
**Hypothetical-1**	5920	2	1032	579					
Track1					0.0022	0.0704	0.0309	2	0
Track2								3	0
**LSU ribosomal protein L17p**	5919	2	618	573					
Track1					0.0042	0.0397	0.1054	1	1
Track2								3	1
**GTP-binding protein EngB**	5923	2	603	603					
Track1					0.077	2.3909	0.0322	0	3
Track2								4	0
**UDP-N-acetylmuramoylalanine--D-glutamate ligase**	5923	3	570	570					
Track1					0.0062	0.2617	0.0237	1	2
Track2					0.0128	0.2843	0.045	0	3
Track3								0	3
**Phospho-N-acetylmuramoyl-pentapeptide transferase**	5923	3	1215	696					
Track1					0.0648	2.6013	0.0249	3	1
Track2								1	3
Track3					0.063	2.4727	0.0255	2	1
**Hypothetical-2**	5923	2	495	495					
Track1					0.0024	0.0334	0.0711	2	1
Track2								2	0
**Phosphoribosylaminoimidazole carboxylase catalytic subunit**	5919	2	477	477					
Track1					0.0078	0.1474	0.0531	1	1
Track2								4	0
**WD40-like Beta Propeller**	5919	2	1752	452					
Track1					0.015	0.2455	0.061	3	0
Track2								4	0
**Alpha,alpha-trehalose-phosphate synthase**	5919	3	1665	1137					
Track1					0.001	0.0245	0.0414	3	0
Track2								4	2
Track3					0.049	0.5309	0.0924	0	2
**Translation initiation factor 1**	5919	2	216	216					
Track1					0.017	0.7127	0.0238	1	1
Track2								3	1
**Hypothetical-3**	5919	4	1164	531					
Track1					0.0002	0.2111	0.001	2	0
Track2					0.0002	0.2434	0.001	1	1
Track3					0	0	0.1903	3	0
Track4								2	1
**Citrate Synthase**	5919	2	1284	840					
Track1					0.0029	0.0914	0.0321	1	1
Track2								2	2
**LSU ribosomal protein L36p**	5919	2	117	117					
Track1					0.0006	0.6391	0.001	1	1
Track2								4	1
**Proteorhodopsin**	5919	2	696	696					
Track1					0	0.0406	0.001	1	1
Track2								2	0

Table S7. Values used as cutoffs for the various BLAST analyses, including a brief description to link with the Methods and Material of the main manuscript.

BLAST Description	Program Used	E-value cutoff	AA %ID cutoff	% Alignment Length Cutoff
F. psychrophilum JIP02 phylogenetic markers against GOM scaffolds	BLASTN	1×10-3	n/a	n/a
Putative CDS against the GenBank NR for rough phylogenetic assignment	BLASTP	1×10-10	n/a	n/a
Putative CDS against *Flavobacteria* sp. MS024-2A	BLASTP	1×10-3	n/a	n/a
Determining CSCGs for pangenome	BLASTP	n/a	80%	80%
Pangenome CSCGs against putative CDS	BLASTP	n/a	80%	80%
Putative peptidases against other bins and pangenome	BLASTP	1×10-5	35%	80%
Putative peptidases against the GenBank NR	BLASTP	1×10-5	35%	80%
Putative CDS of 1 bin against all other bins to determine bin functionality	BLASTP	n/a	40%	80%
