# Supplementary material for: Comparative genomics of planktonic Flavobacteriaceae from the Gulf of Maine using metagenomic data
Source: Microbiome. 2014 Sep 5;2:34. doi: 10.1186/2049-2618-2-34 (PMC4164334; doi:10.1186/2049-2618-2-34)
Supplement: Additional file 3 — Supplemental materials and methods. This file provides the parameters used for selecting 16S rRNA gene fragments, the Celera Assembler, the Geneious Assembler, Geneious SNP detection, and PHYML tree construction. [file 2049-2618-2-34-S3.pdf]

Supplemental Materials and Methods

16S rRNA assignments. The unassembled metagenome was searched with BLASTN for 16S rRNA gene homologs against a database of rRNA sequences [1]. Reads containing at least 250 bp 16S rRNA sequences were subjected to the Ribosomal Database Project (RDP) classifier [2, 3].

Celera Assembler Settings. The following settings were used in the specification file to assemble reads identified in each bin and, subsequently, each sub-bin:

```
utgErrorRate=0.18
ovlErrorRate=0.20
cnsErrorRate=0.20
cgwErrorRate=0.20
merSize=14
doExtendClearRanges=2
doOverlapTrimming=0
doResolveSurrogates=1
doFragmentCorrection=0
utgBubblePopping=1
utgGenomeSize=
merylMemory  = 4000
merylThreads = 2
overlapper = ovl
ovlMemory       = 4GB --hashload 0.7 --hashstrings 60000
ovlThreads      = 2
ovlHashBlockSize = 180000
ovlRefBlockSize  = 4000000
unitigger=bog
cnsConcurrency=6
fakeUIDs = 1
astatLowBound = -20
```

Geneious Assembly and SNP determination Settings. Using Geneious V5.6.2, all reads used to construct scaffolds were trimmed based on quality using an Error Probability Limit set at 0.015 from the 5' and 3' ends of each read. Geneious describes Error Probability Limit as, "Trim bases up until the point where trimming further bases will improve the error rate by less than the limit". The reads for each individual scaffold were then assembled against the scaffold. Geneious settings were the default when Sensitivity = Medium Sensitivity and Fine Tuning = Maximum. Defaults settings were as such:

Allow Gaps Max. per read = 15%

Word length = 14

Ignore words repeated more than 10 times

Max. gap = 50

Index Word Length = 12

Max. ambiguity = 4

Geneious was used to determine possible locations for SNPs. The SNPs identified using this feature were further refined using the method described in Methods and Materials. The setting used were:

Minimum coverage: 2

Minimum Variant Frequency: 0.2

Analyze effect of polymorphisms on translations: Bacterial

Only find SNPs

Don't find variations in annotation types: Editing History Deletion

PHYML, Maximum likelihood phylogenetic tree Settings. PHYML trees were built using the JTT model and a computed using 1,000 bootstraps. The version of PHYML used was part of Geneious V5.6.2 and had the default settings of:

Transition/Transversion ratio: 4 – Estimated

Proportion of invariable sites: 0 – Fixed

Number of substitutions rate categories:1

Gamma distribution parameter: 0 – Estimated

Topology search: NNI

References.

1. Altschul SF, Madden TL, Schäffer AA, Zhang J, Zhang Z, Miller W, Lipman DJ: **Gapped BLAST and PSI-BLAST: a new generation of protein database search programs.** *Nucleic Acids Res* 1997, **25**:3389–3402.

2. Cole JR, Wang Q, Cardenas E, Fish J, Chai B, Farris RJ, Kulam-Syed-Mohideen AS, McGarrell DM, Marsh T, Garrity GM, Tiedje JM: **The Ribosomal Database Project: improved alignments and new tools for rRNA analysis**. *Nucleic Acids Res* 2009, **37**(Database):D141–D145.

3. Wang Q, Garrity GM, Tiedje JM, Cole JR: **Naive Bayesian Classifier for Rapid Assignment of rRNA Sequences into the New Bacterial Taxonomy**. *Appl Environ Microbiol* 2007, **73**:5261–5267.
